# Advancements in Stone Object Restoration Using Polymer-Inorganic Phosphate Composites for Cultural Heritage Preservation

**DOI:** 10.3390/polym16142085

**Published:** 2024-07-22

**Authors:** Toma Fistos, Irina Fierascu, Doina Manaila-Maximean, Radu Claudiu Fierascu

**Affiliations:** 1 National Institute for Research & Development in Chemistry and Petrochemistry—ICECHIM Bucharest, 202 Spl. Independentei, 060021 Bucharest, Romania; toma.fistos@icechim.ro (T.F.); irina.fierascu@icechim.ro (I.F.); fierascu.radu@icechim.ro (R.C.F.); 2 Faculty of Chemical Engineering and Biotechnology, National University of Science and Technology Politehnica Bucharest, 1-7 Gh. Polizu Str., 011061 Bucharest, Romania; 3 Faculty of Horticulture, University of Agronomic Sciences and Veterinary Medicine of Bucharest, 59 Marasti Blvd, 011464 Bucharest, Romania; 4 Faculty of Applied Sciences, National University of Science and Technology Politehnica Bucharest, 060042 Bucharest, Romania; 5 Academy of Romanian Scientists, 3 Ilfov, 050044 Bucharest, Romania

**Keywords:** stone conservation, consolidation, cultural heritage preservation, hydroxyapatite, chitosan, future perspectives

## Abstract

Recent advancements in cultural heritage preservation have increasingly focused on the development and application of new composites, harnessing the diverse properties of their components. This study reviews the current state of research and practical applications of these innovative materials, emphasizing the use of inorganic phosphatic materials (in particular the hydroxyapatite) and various polymers. The compatibility of phosphatic materials with calcareous stones and the protective properties of polymers present a synergistic approach to addressing common deterioration mechanisms, such as salt crystallization, biological colonization, and mechanical weathering. By examining recent case studies and experimental results, this paper highlights the effectiveness, challenges, and future directions for these composites in cultural heritage conservation. The findings underscore the potential of these materials to enhance the durability and aesthetic integrity of heritage stones, promoting sustainable and long-term preservation solutions.

## 1. Introduction

The notion of cultural heritage appeared starting with the 19th century and was defined as the totality of movable or immovable artifacts with historical value. This concept developed slowly and gradually until the middle of the 20th century when society’s perspective on this field changed. With the end of the Second World War, an event in which a significant number of buildings, paintings, sculptures, historical documents, defined as tangible cultural heritage, were partially or totally destroyed, society began to pay more attention to this domain. These factors which are classified as anthropogenic factors combined with the numerous natural factors (strong wind, heavy rain, hurricane, blizzards, drastic temperature changes) which are constantly changing becoming more and more aggressive with the passage of time have led to the emergence of numerous fields with the aim of developing different advanced materials to preserve the objects that belong to the cultural heritage [1].

In society, cultural heritage includes numerous values such as: historical, economic, spiritual, touristic, cultural identity. Probably one of the most well-known roles performed by cultural heritage is represented by its historical importance, which covers a wide area of aspect in society, such as: understanding past societies, preserving history, transmitting knowledge to future generations, reflecting continuity and the evolution of society etc. Cultural heritage provides tangible and intangible evidence of past civilizations, societies and cultures through archaeological sites, artefacts, documents, traditions and oral histories, enabling historians and researchers to reconstruct and understand the lifestyles, beliefs, values and achievements of past societies. By preserving archaeological sites, documents and objects of cultural value that serve as physical memories, the events and milestones that have shaped the history of mankind are kept intact and transmitted to future generations [2].

A second important factor in the preservation of cultural heritage is represented by its economic aspect. Countries that know how to capitalize their archaeological sites, historic buildings and various movable objects with heritage value have managed to develop considerably from an economic point of view. Some significant examples for this purpose are represented by countries such as Mexico, Greece, Portugal, Turkey, Egypt whose economy depends a lot on cultural heritage [3,4]. They managed to develop and prosper thanks to the proper capitalization of their cultural heritage.

An example of a country that took this field beyond the tourist sphere is Italy, where both fundamental and applied research in the field of the development of new materials for the preservation of cultural heritage led not only to the imposition of reference names from a scientific point of view and a whole field of research, but also to the emergence of one of the most important economic agents whose aim is the development and commercialization of such materials [4,5].

In the area of conservation of heritage objects, a series of rules must be respected, and the material to be preserved must be fully understood. The degree of intervention of the object is established following qualitative and quantitative analyses, and the risks and possible damages that the heritage object may suffer as a result of the intervention must always be taken into account. The material selected to conserve the specific heritage object must have physical, chemical, mechanical and aesthetic and chemical compatibility with the support material [6,7], as the main issue in the conservation of heritage objects is to not affect the object itself [8]. Other characteristics that the material must fulfill are: low costs, friendly to the environment, the source of origin is preferably renewable, compatible, easy to apply and easily absorbed by the support material, to allow the exchange of vapors water, to be reversible or retraceable [9,10,11].

Due to natural aging combined with adverse weather, the stone artifacts suffer various degrees of surface damage. In order for them to preserve their original appearance and historical information, different types of materials have been developed to protect them against harmful environmental conditions, biodeteriogens and aging. In order to increase the compatibility of the treatment with the material on which they are applied (stone), the use of nanohydroxides was preferred to prepare uniform dispersions of Ca(OH)_2_, Mg(OH)_2_ and Ba(OH)_2_ that infiltrate the stone and produce carbonates with a protective role [7,12,13]. Another aspect that must be taken into account in the development of a protective material is its hydrophobicity, because water is one of the main factors of damage to stone artifacts. For this purpose, numerous hydrophobic and superhydrophobic polymeric materials have been developed such as: fluorine resin containing nanoparticles of SiO_2_ [14], ZrO_2_ doped with ZnO–Polydimethylsiloxane [15], monomeric and oligomeric ethoxysilanes with SiO_2_ [16], alkyl silicone oil doped with nanoparticles [17,18].

The aim of this review is to critically assess the recent advancements in the development and application of polymer-inorganic phosphate composites, for the restoration and conservation of stone cultural heritage objects. The review seeks to evaluate the effectiveness of these materials in mitigating common deterioration mechanisms, explore the compatibility and integration of inorganic phosphates (particularly hydroxyapatite)/polymers composites with various types of stone, identify current challenges and limitations, and propose future research directions to optimize these conservation strategies for sustainable, long-term preservation of cultural heritage.

Selection of the works to be inserted in the present review was performed using SCOPUS database, as a more exhaustive source of information. The primary selection was performed using the keyword “cultural heritage” (the search providing a total of results), followed by further refining using as search terms within the results “phosphate” (350 documents), “conservation” (275 documents), and “polymer” (112 documents). A secondary selection was made using as document type discriminator “Article”, “Book” and “Book chapter” (70 documents). The resulting documents were further filtered by abstract reading and full-text reading, selection of the works to be included in the presented being based on the clear presentation of inorganic phosphate/polymer composites and their application for the conservation of cultural heritage stone artifacts.

## 2. Currently Used Materials

Over the years, nanoparticles (NPs) have been used in different combinations and concentrations to improve preservation and conservation properties for the desired purpose. One of the important properties in this field is to obtain materials that are as hydrophobic as possible, because water is one of the main factors in the deterioration of cultural heritage objects. One of the most used compounds for this purpose is represented by silicon dioxide NPs (SiO_2_) [19,20,21]; it presents many advantages, including high compatibility with the support material (stone), high stability, in the same time being accessible and inert from chemical point of view. Other composites used to obtain hydrophobic materials with photocatalytic properties were represented by NPs of titanium dioxide (TiO_2_) [22,23] and zinc oxide (ZnO) [24] which were used as and additives in combination with various polymers. Other compounds used as additives after which promising results were obtained are represented by NPs of aluminum oxide (Al_2_O_3_) [25] and tin dioxide (SnO_2_) [26] which were applied to the tiles and increased the surface roughness, thus creating an extremely hydrophobic layer. Another compound with promising results in the conservation of marble or limestone monuments is represented by calcium hydroxide (Ca(OH)_2_) which in combination with atmospheric CO_2_ leads to the formation of calcium carbonate (Ca(CO)_3_) which makes it very compatible with these types of support materials. Due to the low water solubility of (Ca(OH)_2_), it is often used in various colloidal solutions for the preservation of heritage objects made of natural rocks based on calcite [27,28,29].

To be combined with NPs, biodegradable polymers were considered because they present the advantage of complete disintegration while allowing the re-application of the treatment or the application of a new treatment without damaging the treated object [30]. A good candidate in this field is represented by chitosan, as a biodegradable, non-toxic and biosourced polymer (being mostly derived from crustacean remains), obtained from the deacetylation reaction of chitin [31]. Although it fulfills many necessary characteristics in this field, its use is a limited one that needs to be further studied [11]. Several types of polymeric coatings developed for the protection of cultural heritage were recently presented by our group [32].

## 3. Application of Polymer-Inorganic Phosphate Composites in Conservation Procedures

Inorganic phosphates play a crucial role in the consolidation of stone cultural heritage objects, offering significant benefits in preserving and restoring these valuable artifacts. One of the primary advantages of using phosphate salts is their ability to chemically interact with the stone substrate, leading to the formation of new, stable mineral phases, such as calcium phosphate, which can enhance the durability and structural integrity of the stone (usually calcium phosphates, particularly hydroxyapatite) [33]. Unlike organic consolidants that may deteriorate over time or become visually intrusive, inorganic phosphates form permanent, non-visible bonds with the stone, ensuring a more lasting and aesthetically acceptable solution [33,34,35].

Moreover, inorganic phosphates are particularly effective in mitigating the effects of weathering and deterioration caused by environmental factors such as acid rain, freeze-thaw cycles, and biological growth. They can penetrate deeply into the stone, reinforcing it from within and reducing its porosity, which helps to prevent further ingress of water and pollutants [34,35]. This deep penetration also means that the treated stone retains its natural appearance and breathability, essential for the preservation of its original characteristics. In the last years, the application of hydroxyapatite (a form of calcium phosphate) for the consolidation and protection of historical stone gain attention and scientific support, as recently presented in several review works [7,36]. As previously presented by our group [37], the inorganic phosphatic materials (and, particularly, hydroxyapatite), represents a scientifically sound and sustainable approach to preserving historical and culturally significant structures for future generations. In the same time, considering the advantages of the most recent developments regarding the polymeric coatings for cultural heritage objectives [32,38], as well as in the larger area of organic/inorganic composites [39], which were proven to be effective in electro-optical applications [40,41], and even the conservation of paper with cultural value [42], the need for a next generation composite material for the protection of stone objects with cultural value, harvesting the advantages of the two polymeric phase and of the phosphatic (apatitic) phase, clearly arises.

The approach would offer substantial advantages: one of the key benefits is represented by their ability to enhance the mechanical properties of degraded stone materials. Inorganic phosphates (particularly hydroxyapatite—HAP, either formed in-situ, as a result of the interaction between phosphate salts and the stone material, or added as an ex-situ formed material) provide excellent structural stability and compatibility with calcareous stones. When incorporated into a polymer matrix, the inorganic phase reinforces the composite, making it capable of withstanding environmental stressors such as thermal expansion, freeze-thaw cycles, and mechanical impacts. This reinforcement ensures the long-term durability and stability of the treated stone, thereby preserving its structural integrity and historical value [36]. Furthermore, the bioactivity and chemical affinity of hydroxyapatite with carbonate stone materials facilitate a seamless integration and consolidation process. HAP can chemically bond with the calcium carbonate in the stone, forming a coherent and compatible interface that strengthens the stone from within.

The polymer matrix, meanwhile, enhances the workability and application of the composite, allowing an uniform and homogeneous spread of the composite. Additionally, hydroxyapatite-polymer composites are typically transparent or minimally altering in appearance, ensuring that the aesthetic and historical authenticity of the stone object is preserved [34,43]. This combination of mechanical reinforcement, chemical compatibility, and aesthetic preservation makes hydroxyapatite-polymer composites an advanced and effective solution for the conservation of cultural heritage stone objects.

**Figure 1:**
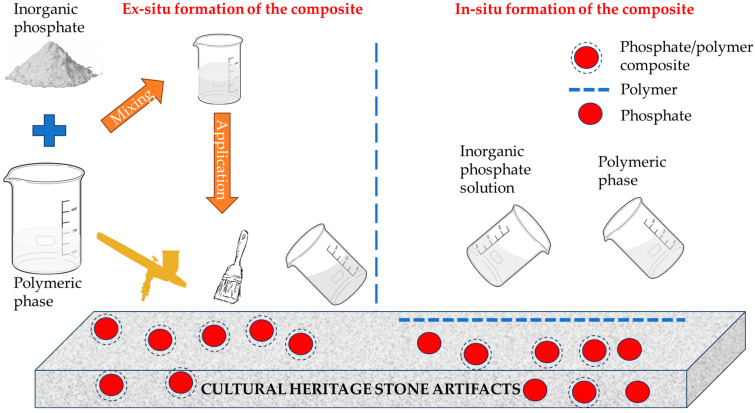
Schematic depiction of the two methods for developing inorganic phosphate/polymer composite for stone conservation.

Considering the two phases with different purposes and properties, two different application methods also arise: the development of composites, followed by their application on the envisaged objectives, respectively the subsequent treatment with the separate materials, with the in-situ formation of the composites (Figure 1). The two methods, with case studies regarding their use will be presented in the following section.

## 4. Case Studies

### 4.1. Ex-Situ Composite Development

#### 4.1.1. Composite Development and Application Methods

The ex-situ formation of inorganic phosphates/polymer composites involves a meticulously controlled synthesis process, in multiple steps. The approach, although more laborious, ensures the creation of a material with optimal properties for reinforcing and protecting stone substrates. The procedure generally comprises several critical steps, including the preparation of the phosphatic material, the synthesis of the polymer matrix, and the integration of both components into a composite material.

The selection of the polymer matrix represents a critical step, often being used polymers such as poly(methyl methacrylate) (PMMA) or polyvinyl alcohol (PVA), known for their compatibility with stone and ease of processing [44]. The polymer is dissolved in an appropriate solvent to create a homogenous polymer solution.

The integration of the phosphatic phase into the polymer matrix is achieved by dispersion of the inorganic powder into the polymer solution, ensuring thorough mixing to achieve a uniform distribution of the inorganic particles within the polymer matrix. This mixture is then subjected to processes such as sonication or mechanical stirring to break up any agglomerates and ensure a homogenous composite [45].

Finally, the composite material is applied to the stone substrate. The application can be performed using techniques such as brushing, spraying, or injecting, depending on the stone’s porosity and the extent of deterioration. Upon application, the solvent evaporates, leading to the formation of a solid composite that penetrates the stone pores and consolidates its structure. The resultant composite material should ideally provide enhanced mechanical properties, reduced porosity, and improved resistance to environmental degradation, ensuring the long-term conservation of the cultural heritage stone.

#### 4.1.2. Hydroxyapatite/Polyelectrolytes Composites

The application of a composite material made of polyelectrolytes and hydroxyapatite nanocrystals for the protection and restoration of calcareous cultural heritage stones is presented by Hafez and Biskos [46]. The study addresses the challenges associated with the deterioration of calcareous stones, which are prevalent in many historical monuments and artifacts. The authors describe a detailed synthesis process for hydroxyapatite nanocrystals, directly in the branched polyethylenimine (PEI) matrix. An interesting point in the study is represented by the evaluation of phase composition and morphology of the inorganic phase with PEI content. Depending on the PEI content, secondary phases, such as dicalcium phosphate dehydrate or amorphous calcium phosphate can be observed, with variable dimensions. The resulting nanoparticles were resuspended in two different solvents (ethanol and water, containing 40 mM PEI) for application by spraying. The treatment itself was performed using two different marble models (with different grain sizes) and involved the pre-treatment with the polyelectrolytes (polyacrylic acid—PAA) and PEI, applied by layer-by-layer deposition, chosen for their ability to protect the marble from further deterioration and to serve as a intermediate bonding layer for the composite) followed by the application of the HAP/PEI composite by spray-coating, and, finally an outer layer consisting of PAA.

Experimental results demonstrate that the pre-treatment significantly enhances the mechanical strength and durability of the calcareous stone, registering an increased acid resistance (46% decrease in mass loss compared with untreated sample) and a decreased water absorption (39% compared with the control), as well as a slight increase in the hydrophobicity. Additionally, the composite material shows excellent compatibility with the stone, ensuring that the treatment does not alter the stone’s appearance (color difference ΔE * < 5, in the case of fine-grained marble), while presenting a smooth uniform and densely packed coating with further protective role, with some minor cracks appearing for the use of ethanol as a solvent, due to its much faster evaporation (compared with water). The study concludes that the use of polyelectrolytes and hydroxyapatite nanocrystals (crystal size < 30 nm) offers a promising solution for the conservation of calcareous cultural heritage stones, combining the benefits of modern materials science with the need for historical preservation.

#### 4.1.3. Sodium Tri-Polyphosphate/Chitosan Composites

Sodium tri-polyphosphate (TPP), another inorganic phosphate commonly used for conservation purposes, was evaluated in an inorganic/polymeric composite for the protection of stone by Silva et al. [11]. The study focused on formulating chitosan/TPP-based coatings and evaluating their effectiveness in preventing microbial growth on stone surfaces. Chitosan, a natural biopolymer derived from chitin, is chosen for its biocompatibility, biodegradability, and inherent antimicrobial properties, while TPP was used to enhance the mechanical properties and stability of the chitosan coating. The chitosan/TPP coatings were prepared by mixing chitosan solutions with TPP under controlled conditions to form a stable gel. These coatings were then applied to stone samples commonly used in outdoor sculptures (granite, marble and limestone). The treated stones were subjected to accelerated aging tests, including exposure to microbial cultures, to simulate real-world environmental conditions.

The coatings (evaluated as solid films) showed strong antimicrobial activity against a range of microorganisms commonly associated with biodeterioration, including bacteria (*Pseudomonas aeruginosa*, *Staphylococcus aureus*, *Bacillus cereus*), and fungi (*Penicillium chrysogenum,* and, of particular importance, the pigment-producing *Rhodotorula* spp.). This activity was attributed to the combined effects of chitosan’s antimicrobial properties and the structural stability provided by TPP crosslinking. Also, the solid films had an increased solubility in water (most probably due to the presence of more unbound polar groups with TPP content increase, which could interact with water molecules), and a decreased swelling degree (most probably due to the increase in the crystallinity degree of chitosan with the higher TPP content, which would lead to a reduced chain mobility and water affinity). Consistent with the solubility assays, the water contact angle decreased with the increase in TPP content (although significant differences were only recorded between the extreme TPP levels) and to an increase in the permeability of the films (although the increase was only observed up to chitosan: TPP mass ratio = 1, after which a decrease in the permeability was observed). The decrease in permeability for higher TPP content was assigned by the authors to the reduction in intermolecular distances due to TPP agglomeration.

Importantly, the application of the coatings did not adversely affect the physical and aesthetic properties of the stone. The treated stones retained their natural appearance, color, and texture, which is crucial for preserving the visual integrity of cultural heritage objects, lower ΔE* values being recorded for the application by brushing (<1 for granite and limestone, 2.25 for marble), compared with the application by micropipette (approx. 1 for limestone, above 2 for granite and marble). In the same time, the composites reduced the wettability of the stones for the granite and limestone samples, although not acting as a water-repellent.

The surface evaluation of the treated samples revealed an uneven distribution of the composites, which the authors assign to both natural adsorption into the pores of the stones, as well as to the application method (by pipetting), which led them to the proposal of other application alternatives for the in-situ application (by brushing or spraying).

The main conclusion of the study is represented by the proposal of the chitosan/TPP-based coatings as an effective and environmentally friendly solution for the conservation of outdoor stone sculptures, considering both the in vitro assays and the results of the test on model stone samples. By preventing microbial colonization and enhancing the stone’s resistance to environmental stressors, these coatings can significantly extend the lifespan and preserve the aesthetic and historical value of cultural heritage objects.

As a general conclusion of the ex-situ approach, the composite appears to provide a decrease in wettability, providing protection against several environmental factors. The pre-treatment using a polymeric solution tend to provide a more uniform coating, compared with the sole application of the composite. The polymerization of the organic phase of the composite was presumed to take place, in both presented case-studies, both on the surface and on the pores of the stone. This, in turn, raises some questions regarding the long-term influence of the composites on the stone’s intrinsic properties, which should be further studied.

### 4.2. In-Situ Composite Development

#### 4.2.1. Major Differences between the In-Situ and Ex-Situ Approaches

The in-situ formation of the inorganic phosphate and polymer composites involves synthesizing the composite directly within the stone matrix, as opposed to the previously discussed ex-situ approach, where the composite is formed externally and then applied to the stone. Both methods aim to enhance the stone’s structural integrity and resistance to environmental degradation, but they differ in terms of their application procedures and interaction with the stone substrate. Often, the in-situ approach is performed in two steps, in the first one being applied the inorganic phase, followed by the polymeric treatment.

Both in-situ and ex-situ formation processes aim to enhance the stone’s structural integrity, but they offer different advantages and challenges. The ex-situ method allows for precise control over the composite’s properties before application, ensuring uniformity and consistency. However, the depth of penetration may be limited, and the interaction between the composite and the stone may not be as intimate as desired. Conversely, the in-situ method can achieve deeper penetration and a more integrated formation of the composite within the stone’s pore structure, potentially resulting in better mechanical reinforcement and durability. However, in-situ processes require careful control of reaction conditions within the stone, which can be challenging to achieve uniformly.

#### 4.2.2. The Use of Diammonium Hydrogen Phosphate as HAP In-Situ Precursor

Diammonium hydrogen phosphate (DAP) represents one of the most recent additions on the list of accepted restoration and conservation materials for carbonaceous stones. The treatment with DAP solutions enhances the durability of weathered calcareous stones by restoring their lost cohesion, the mechanism being based on the formation of the consolidating agent (calcium phosphate) by the reaction of DAP with neighboring calcium carbonate (from the stone structure) [36], following reaction (1):

(1) $$5{\left({NH}_{4}\right)}_{2}H{PO}_{4}+10{CaCO}_{3}\to {Ca}_{10}{{(PO}_{4},{CO}_{3})}_{6}{\left(OH,{CO}_{3}\right)}_{2}+5{\left({NH}_{4}\right)}_{2}{CO}_{3}+3{CO}_{2}+2H_{2}O$$

Andreotti et al. [47] evaluated effectiveness of DAP/polymer composites-based treatment designed to prevent damage caused by salt crystallization in stone materials. Salt crystallization is a significant deterioration mechanism for stone used in cultural heritage, leading to mechanical stress, flaking, and eventual loss of material integrity.

The polymers tested included poly(acrylic acid), alginic acid, tannic acid, and chitosan, while the inorganic DAP phase was applied as pre-treatment of the limestone samples, acting, besides its consolidant properties, as anchoring sites for the polymers. Both steps of the treatment procedures were performed by immersion of the stone samples in treatment solutions, the results suggesting superior effects for the two-phase treatment compared with the use of polymeric treatment alone.

The visual appearance of the samples following the treatment was negatively influenced by the treatment with poly(acrylic) acid without DAP pre-treatment and especially by the treatment with tannic acid (with or without pre-treatment).

Stone samples treated with both phases exhibited significantly lower weight loss compared to samples subjected to polymer treatment or untreated samples. Interestingly, the DAP alone also reduced the weight loss of the samples. The effect of the DAP pre-treatment was assigned by the authors to several process, acting synergistically: enhancement of the stones’ tensile strength, facilitation of capillary flow towards the surface by the porous treated surface, thus reducing the subfluorescence, as well as the prevention of calcite dissolution. The best results were obtained for the samples treated with DAP and chitosan (12.3% mass loss, compared with 16.7% for chitosan treatment, and 15.2% for untreated sample). Similar results were obtained for salt crystallization under continuous capillary absorption tests (a more relevant assay), in which the DAP pretreatment followed by chitosan treatment reduced the mass loss with approx. 93%, compared with untreated stone (significantly higher than the DAP pre-treatment or the polymeric treatment alone), suggesting a synergistic effect of the two phases. Good results (reduction of 78%, and, respectively, 83%) were also obtained for the DAP pre-treatment followed by poly(acrylic acid), and alginic acid, respectively.

The treatment did not significantly influence the adsorption of the saline solution or the dynamic elastic modulus in any of the experimental variants. The study concludes that the new polymer-based treatments are highly effective in mitigating salt crystallization damage in Globigerina limestone. The treatments (in particular the DAP/chitosan treatment) offer a robust solution for the preservation of stone in cultural heritage applications, not only enhancing the durability and longevity of stone materials but also ensuring the maintenance of their aesthetic and historical values [47]. The authors also concluded that the DAP pre-treatment leads to an improvement of polymer adhesion to the pore surface, while the mechanisms behind the polymeric phase action can be summarized as minimizing disjoining pressure (for poly (acrylic acid) and alginic acid), respectively its action as crystallization inhibitor (for the case of chitosan).

A similar approach was presented by Bassi et al. [48], who further investigated the DAP/chitosan-based treatment aimed at mitigating the effects of salt-induced deterioration in cultural heritage materials (using as test samples two kinds of porous limestones—Globigerina limestone and Lecce stone).

The study constructed on the conclusions of the previously presented study [47], proposing the use of DAP as pre-treatment (in a formulation containing 0.1 M DAP, 0.1 mM CaCl_2_ in 10% ethanol, harvesting its consolidating and polymer-anchoring role) followed by the treatment with 0.05 wt.% chitosan solution, both phases being applied by capillarity.

The authors determined several parameters of the stone samples, before and after treatment (bulk density, open porosity and dynamic elastic modulus), as well as the influence of the applied treatment on the mechanical and physical properties of the stones following accelerated salt crystallization tests (wetting-drying cycles, respectively continuous capillary absorption of a saline solution). According to the results presented by the authors, the treatment led to a systematic minor increase in the dynamic elastic modulus, showed similar porosity and pore size distribution, although the treatment led to a significant decrease in capillary water adsorption coefficients (being registered reduction in the adsorption coefficient of more than 67% for Lecce stone, respectively more than 76% for Globigerina stone, for the inorganic/organic treatment, compared with the untreaded sample), accompanied by a minor reduction of the total water adsorption after 24 h, registered for all the experimental variants.

The salt crystallization tests were performed on different shaped stone samples. The salt crystallization cycles in sodium sulphate solution, performed on cylindrical stone samples, revealed the improvement of the stones’ resistance, determined as sample weight loss, the most efficient treatment, for both types of stones, being the combined chitosan/HAP treatment, for which a reduction of mass loss of approx. 84% for Lecce stone, respectively 69% for Globigerina limestone, compared with untreated samples, were recorded. The treatment also did not negatively influence the mechanical properties of the samples, the combined treatment leading to a slight increase of the tensile strength.

The second salt crystallization test (the capillary absorption of sodium sulphate solution) was performed on prismatic stone samples. The results obtained confirmed that the combined treatment had superior performance over individual compounds (the results obtained being similar to the first test), while, in terms of mechanical properties, the combined treatment led to a significant increase in the dynamic elastic modulus value, even when compared with unweathered stone (for the Globigerina limestone).

The process by which the composites act as a consolidating treatment is assigned by the authors to the improvement of the tensile strength of stone and efflorescence favoring by the formed phosphate (HAP), which also act as chitosan anchoring sites and prevents calcite leaching.

The study concluded that the biopolymeric/inorganic phosphate treatment significantly enhances the durability of tested stones against salt-induced deterioration, offering a promising solution for the conservation of cultural heritage materials. Its compatibility with historical substrates and environmental friendliness making it suitable for widespread application in heritage conservation.

Table 1 summarizes the characteristics of the composite materials described in the previous paragraphs, as well as the main findings of the studies.

**Figure 2:**
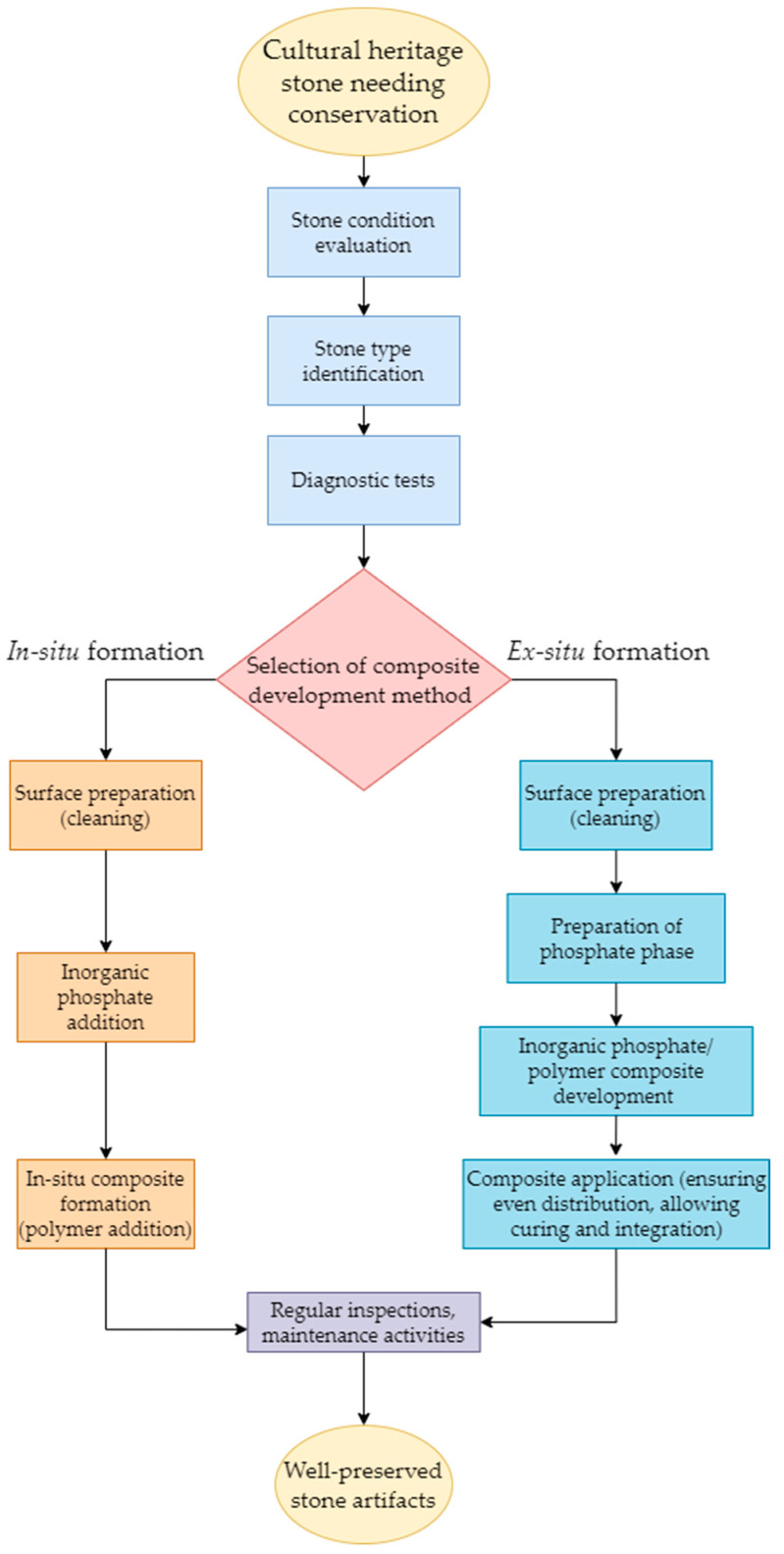
Flowchart of the two conservation methods.

The flowchart of the two different conservation approaches is presented in Figure 2.

## 5. Lessons Learned and Implications of the Present Study

The application of inorganic phosphates (particularly hydroxyapatite)/polymers composites for the restoration and conservation of stone in cultural heritage has yielded significant insights and lessons over recent years. These composites have demonstrated considerable potential in enhancing the durability and preserving the aesthetic and structural integrity of heritage stone materials. However, their application also reveals certain limitations and challenges that must be addressed to optimize their effectiveness.

One of the main lessons learned is the importance of selecting materials that are compatible with the specific type of stone being treated [36]. Several studies revealed that hydroxyapatite, due to its chemical similarity to the minerals found in calcareous stones, integrates well and provides substantial reinforcement without altering the stone’s appearance or physical properties [36,37,43,46]. This compatibility is crucial in ensuring that the conservation treatments do not inadvertently cause further damage or discoloration.

Effective penetration of the composite into the stone’s pore structure is critical for long-term durability. Research indicates that in-situ formation of HAP within the stone matrix often achieves more uniform distribution of the composite compared to ex-situ methods (which requires a polymeric phase pre-treatment for ensuring a uniform coating [46]). Ensuring uniform distribution within the stone’s porous network enhances the mechanical strength and resistance to environmental factors such as water ingress and salt crystallization.

The use of biocompatible and environmentally friendly materials, such as biopolymers (i.e., chitosan, which was proven to be an effective crystallization inhibitor) combined with inorganic phosphates (i.e., DAP or HAP), has been recognized as beneficial. These materials minimize the ecological footprint of conservation activities and reduce health risks to conservators and the public [48]. Moreover, they align with the broader sustainability goals in heritage conservation.

One of the main limitations in applying these composites is the complexity and precision required in their application. In-situ formation processes necessitate precise control over environmental conditions such as pH, temperature, and concentration of precursor solutions, which can be challenging to achieve uniformly in large-scale or outdoor settings. Additionally, ensuring thorough and uniform penetration across irregular stone surfaces can be difficult.

While hydroxyapatite and certain polymers exhibit excellent properties for stone conservation, their performance can vary depending on the specific type of stone and environmental conditions. For example, the efficacy of HAP in non-calcareous stones is still under investigation, and its long-term behavior in different climatic conditions needs further study. Similarly, some polymers may degrade or lose effectiveness when exposed to UV radiation, moisture, or temperature fluctuations.

The cost of materials and the labor-intensive nature of these treatments can be prohibitive, especially for large-scale conservation projects. Developing more cost-effective formulations and application techniques is essential to make these treatments accessible for a broader range of heritage sites.

A significant challenge lies in the non-destructive assessment and monitoring of treated stones. Advanced techniques such as micro-CT scanning, spectroscopy, and other non-invasive methods are necessary to evaluate the penetration, distribution, and effectiveness of the treatments without damaging the stone. However, these technologies can be expensive and require specialized expertise. Also, long-term studies are necessary to assess the longevity of these treatments under varying environmental conditions.

In conclusion, the application of inorganic phosphates/polymer composites shown promising results in the conservation of cultural heritage stones. Lessons learned highlight the importance of material compatibility, effective penetration and environmental considerations. However, challenges such as application complexity, material limitations, economic constraints, and the need for advanced monitoring techniques and long-term performance evaluation remain. Addressing these limitations through ongoing research, technological advancements, and interdisciplinary collaboration will be crucial for optimizing these conservation strategies and ensuring the sustainable preservation of cultural heritage.

The use of composites developed using inorganic phosphates (the most studied one being the hydroxyapatite—HAP) and polymers for the restoration and conservation of stone in cultural heritage has far-reaching implications. These advanced materials offer a multi-faceted approach to conservation, providing both mechanical reinforcement and protection against environmental degradation. Hydroxyapatite’s chemical compatibility with calcareous stones makes it an ideal consolidant, effectively filling cracks and pores to improve structural integrity without compromising the stone’s natural appearance or historical value.

## 6. Conclusions and Future Perspectives

When using inorganic phosphates such as HAP and biopolymers (such as chitosan), the biocompatibility and environmental friendliness of these materials are significant advantages, aligning with the increasing emphasis on sustainable conservation practices. The non-toxic nature of hydroxyapatite and certain biopolymers ensures that their application does not pose health risks to conservators or the public and does not adversely affect the environment. This makes them suitable for in-situ applications, even in sensitive areas where traditional chemical treatments might be too harsh or damaging.

Furthermore, the application of these composites can significantly extend the lifespan of heritage stones by protecting them from common deterioration mechanisms such as salt crystallization, biological colonization, and mechanical weathering. This not only preserves the aesthetic and historical value of cultural heritage but also reduces the frequency and cost of maintenance and restoration interventions. By enhancing the durability of heritage structures, these treatments contribute to their sustainable management and conservation.

Future research should focus on optimizing the properties of the composites to enhance their effectiveness across a wider range of stone types and environmental conditions. This includes tailoring the particle size, surface properties, and concentration of the inorganic phase to maximize its penetration and bonding within different stone matrices. Additionally, the development of new polymers or polymer blends that offer improved UV resistance, flexibility, and durability will be crucial for expanding the applicability of these treatments.

Long-term studies are needed to better understand the performance of these composites under various environmental conditions. This includes assessing their resistance to UV radiation, temperature fluctuations, moisture, and pollution over extended periods. Such studies will provide valuable data on the longevity and effectiveness of the treatments, helping to refine application techniques and material formulations. Monitoring treated structures over decades, rather than years, will offer insights into potential degradation mechanisms and inform future conservation strategies.

Innovations in application techniques are essential to ensure the uniform and deep penetration of these composites into the stone. Research should explore non-invasive and minimally invasive methods that can deliver the treatment more effectively, such as advanced spraying systems, micro-injection techniques, or the use of nanotechnology. These methods could help achieve more consistent results, particularly on complex or heavily weathered surfaces.

The integration of digital technologies such as 3D scanning, modeling, and AI-driven diagnostics can revolutionize the application and monitoring of these treatments. Digital tools can help assess the condition of heritage stones with high precision, identify areas requiring treatment, and simulate the effects of different conservation strategies. This data-driven approach can optimize the application process, ensuring that treatments are precisely targeted and adapted to the specific needs of each structure.

Finally, research should focus on developing scalable and cost-effective solutions to make these advanced materials accessible for large-scale conservation projects. This includes exploring the use of locally sourced raw materials, improving the efficiency of synthesis processes, and developing simplified application protocols that can be implemented by conservation practitioners without specialized equipment. Ensuring that these innovative treatments are both affordable and practical for widespread use is essential for their adoption across diverse heritage sites worldwide.

## Figures and Tables

**Table 1:** Characteristics of the inorganic phosphate/polymeric composites and their main effects in the conservation of stone artifacts (case-studies presented in chronological order).

Composite Formation Approach	Inorganic Phase Properties	Polymeric Phase Properties	Composite Characteristics	Main Findings	Ref.
Ex-situ	HAP synthesized in the polymer matrix from calcium chloride and dipotassium phosphate 20 mM, crystal size < 30 nm	Branched polyethylenimine (PEI, 40 mM, MW750,000, pH = 9.5)	Mass to solvent ratio for application 1:10, pH for water solvent = 6.2, no pH correction for ethanol. Treated stones—acid resistivity, quasi-static contact angle, water adsorption and color alteration	At a concentration of 300 mg/L PEI, HAP is the major phase, with round-shaped crystals; the polyelectrolyte pre-treatment provided increased acid-resistance properties and a reduction of the water adsorption capacity, while the composite treatment (using water as solvent) formed an uniform coating, and reduced the color variation of the polyelectrolyte pre-treatment	[46]
	Commercial sodium tripolyphosphate, used at 0.25%, 0.50% and 0.75%	Chitosan, medium molecular weight grade (190,000–310,000 Da), 75–85% deacetylated, cross-linked with citric acid, 1%	6 mL TPP solution added dropwise into 15 mL polymer solution, thoroughly mixed; characterized using FTIR, swelling in water, wettability, thickness and water vapour transmission rate and antimicrobial properties	The appropriate solution for stone treatment—0.25%TPP, following in vitro tests; lowest color variation observed on application by brushing; composite partially polymerized on the surface of the stone; decreased wettability for coating granite and limestone; negative effect on the wettability of marble	[11]
In-situ	Solution 0.1 M DAP (commercial) + 1 mM CaCl_2_ + 10% ethanol in water	Poly(acrylic acid), sodium salt (PAA, Mw = 2100), alginic acid, sodium salt (from brown algae, low density, low viscosity, ALA), tannic acid (TA), chitosan (from shrimp shells, low viscosity)	The tested limestone was subjected to pre-treatment with DAP solution, followed by the treatment with 0.5%PAA, 0.2%ALA, 0.01%TA, respectively 0.05%chitosan by partial immersion. Stone samples were subjected to salt crystallization tests	TA led to significant color variations and was not used in further tests; DAP pre-treatment led to an increase in salt resistance; Multiple cycles salt resistance: dry scaling observed at cycle 1 for PAA, cycle 2 for untreated sample and DAP, cycle 3 for samples DAP/PAA, ALA and chitosan treatments, cycle 4 for samples DAP/ALA and DAP/chitosan.	[47]
	Solution 0.1 M DAP (commercial) + 1 mM CaCl_2_ + 10% ethanol in water	Chitosan	The tested limestone was subjected to pre-treatment with DAP solution, followed by the treatment with 0.05% chitosan by partial immersion. Stone samples were subjected to water adsorption tests, tensile splitting tests, and salt crystallization tests	Limited (insignificant) weight increase after treatment; systematic increase of dynamic elastic modulus; treatments promoted formation of efflorescence, and improved the stone resistance (best results obtained for HAP/chitosan treatment–weight loss decrease 84%—Lecce stone, 69%—Globigerina limestone); no negative influence on tensile strength after salt crystallization cycles.	[48]

## Data Availability

Not applicable.
